# Offered Support and Knowledge about the Menstrual Cycle in the Athletic Community: A Cross-Sectional Study of 1086 Female Athletes

**DOI:** 10.3390/ijerph191911932

**Published:** 2022-09-21

**Authors:** Philip von Rosen, Linda Ekenros, Guro Strøm Solli, Øyvind Sandbakk, Hans-Christer Holmberg, Angelica Lindén Hirschberg, Cecilia Fridén

**Affiliations:** 1 Department of Neurobiology, Care Sciences and Society, Division of Physiotherapy, Karolinska Institutet, Alfred Nobels Allé 23, 141 52 Huddinge, Sweden; 2 School of Sport Sciences, UiT—The Arctic University of Norway, 9019 Tromsoe, Norway; 3 Department of Sports Science and Physical Education, Nord University, 8026 Bodø, Norway; 4 Department of Neuromedicine and Movement Science, Centre for Elite Sports Research, Norwegian University of Science and Technology, 7034 Trondheim, Norway; 5 Department of Health Sciences, Lulea University of Technology, 971 87 Lulea, Sweden or; 6 Department of Physiology and Pharmacology, Biomedicum C5, Karolinska Institutet, 171 77 Stockholm, Sweden; 7 Department of Women’s and Children’s Health, Karolinska Institutet, 171 76 Stockholm, Sweden or; 8 Department of Gynecology and Reproductive Medicine, Karolinska University Hospital, 171 76 Stockholm, Sweden

**Keywords:** amenorrhea, hormonal contraceptives, menstrual cycle, physical performance, sport

## Abstract

Many female athletes perceive that symptoms related to the menstrual cycle such as dysmenorrhea, premenstrual symptoms, amenorrhea or side-effects of hormonal contraceptives negatively impact their training, performance, and general well-being. Knowledge and communication about female athletes’ health is therefore important in the sport community. The aims of this study were to explore the level of knowledge and communication about menstrual cycle issues and use of hormonal contraceptives in the athletic community and to describe the kinds of medical support offered to female athletes. A total of 1086 Swedish and Norwegian athletes from 57 different sports responded to a web-based questionnaire. Of these, 58% (*n* = 627) practiced team sports and 42% (*n* = 459) individual sports. Twenty-six percent (*n* = 278) of the athletes perceived their knowledge about female athlete health to be poor/very poor and the knowledge was most often acquired from medical staff. Fifty-three percent (*n* = 572) of the athletes perceived the knowledge acquired of their coaches as poor/very poor, even though a significantly (*p* < 0.001) higher proportion of athletes with a female coach (30%, *n* = 31) rated their coach’s knowledge as very good/good, compared to athletes with a male coach (5%, *n* = 31). Only 11% (*n* = 116) of the athletes discussed female health issues with their coach. The majority (81%, *n* = 842) of the athletes partly to strongly agreed that female athlete health is considered a taboo topic in the athletic community. Forty-seven percent (*n* = 510) of the athletes had access to a physiotherapist, while only three percent (*n* = 29) had access to a gynecologist. Low perceived knowledge, lack of communication and support demonstrate the need for a multi-professional medical team and enhanced educational efforts focused on female athlete health in the athletic community.

## 1. Introduction

A high proportion of female athletes perceive that symptoms related to the menstrual cycle (MC) impact their training, performance, and general well-being [1,2,3,4,5,6]. For instance, 71–83% of female athletes experience dysmenorrhea, associated with symptoms such as abdominal or back pain/cramps, headache, diarrhea and nausea during the first days of menses that likely affect sports participation and performance [1,2,4,5,7]. During the late luteal phase, premenstrual symptoms (PMS) with varying severity, such as breast tenderness, bloating, headache, irritability, depression, tearfulness, and fatigue, have been reported to affect performance in female athletes [2,4]. However, a low proportion of coaches and athletes seem to consider these common symptoms when planning training and competition [2]. The communication and support provided to handle these symptoms and their possible effects on training and performance is not well explored in female athletes.

Menstrual disturbances, including amenorrhea and oligomenorrhea, are common among athletes, although the prevalence varies widely between sports and is often related to extensive training and/or relative energy deficiency (RED-S) [8,9,10,11]. Although the combination of extensive training, RED-S and menstrual disturbances, often referred as the female athlete triad, could lead to short- and long-term consequences [12], many coaches and athletes consider this triad to be acceptable or, indeed, even a requirement for elite performance by female athletes [13]. This indicates a lack of knowledge among coaches and athletes.

Hormonal contraceptives (HCs) are commonly used by female athletes [2,4] even if the effect of exogenous female sex hormones on physical performance is not fully explored [14]. HCs are mainly used for contraception but also for treatment of dysmenorrhea, menorrhagia, PMS, amenorrhea and to postpone or omit menstrual bleeding [14]. Athletes’ and coaches’ knowledge about different HCs, possible side-effects and impact on training and performance are so far not extensively studied. A recent qualitative study shows that knowledge and understanding of the MC varies in female coaches, with personal experience impacting the information and support provided to athletes [15].

A high proportion of female athletes perceive that both different aspects of their MC and HCs influence their training and performance. Still, they do not feel comfortable discussing these issues with their coaches [1,2,5,13]. General knowledge about the MC and HCs in relation to training and performance has been reported to be low among both athletes and their coaches [1,2,5,13,16,17]. Interpersonal and structural barriers have been reported to hinder communication [13]. Feeling uncomfortable or unexperienced discussing female health with their coach, and lack of formal or organized forums and multidisciplinary teams were described as such barriers [13]. A strong coach–athlete relationship based on trust and communication is very important for athletes, not least for success in elite sports [18].

In fact, even many members of medical personnel may vary in their knowledge about the different aspects and treatment of the female athlete triad [19,20]. To improve this situation, it is necessary to determine the kinds of support through coaches and medical staff that is currently available to female athletes, as well as to identify from where athletes seek their support. By exploring these aspects, an increased understanding of the female athlete might help the development of different support functions.

Accordingly, the primary aims of this study were (a) to explore the level of knowledge and communication about the MC, MC-related disturbances and use of HC in the athletic community and (b) to describe the support offered to female athletes. A secondary aim was to evaluate potential differences in these respects between athletes competing at different levels and of different ages.

## 2. Methods

### 2.1. Study Population and Distribution of the Survey

Female athletes at least 18 years old and active members of the Swedish or Norwegian Sports Federation were recruited via social media and through emails to professional sports clubs. As a result, 1086 participants in 57 different sports consented to participate in the study (Table 1). All received comprehensive information about the study before providing their written consent prior to completing the questionnaire. This study was approved by the Swedish Ethical Review Board (Dnr 2020-00418).

### 2.2. Design and Distribution of the Survey

A questionnaire was developed and designed to explore demographic data, MC history, perception of athletic performance and support provided to the athlete. The questionnaire was originally written in Swedish and later translated into Norwegian by a native speaker. Based on a pilot study involving 124 female athletes (median age 20 (IQR 19–24 years), median Body Mass Index (BMI) 22.2 (IQR 21.2–23.5)), minor modifications of the questions were made. Between June and December of 2021, the modified questionnaire, consisting of 46 questions and requiring approximately 20 min to complete, was filled in online by the athletes. Six of the questions were closed-ended, and forty of the questions had multi-select-answer options. Written responses were only requested when the answer chosen was “Other”. Based on the pilot study, face validity and content validity have been found to be satisfactory and internal consistency, related to items that measures the same construct, as adequate (Cronbach’s alpha, 0.62–0.74).

The athletes reported demographic information, MC history, potential effects of the MC on athletic performance, use of HCs, support provided to the athlete and knowledge about “female athlete health”. In this study, female athlete health refers to how the female sex hormones during the MC, and during use of HCs, affect the female athlete’s body. Only the parts of the questionnaire about demographic data, support provided to the athlete and knowledge about female athlete health were used in this study. The full questionnaire is available through the authors.

### 2.3. Statistical Analysis and Data Categorization

The data were screened for duplicates, and the questionnaires with substantial missing data (*n* = 23) were removed and not included in the analysis. The 1086 included athletes were categorized as top-elite athletes (i.e., athletes participating in the Olympic games, World or European championships; 15%, *n* = 158), elite athletes (i.e., athletes participating in other international competitions, national championships, or the highest national league; 36%, *n* = 393) and sub-elite athletes (i.e., athletes participating in the second highest national league or district competitions; 49%, *n* = 535), in a variable called athlete level. Sports were divided into individual and team sports. Differences in background characteristics of the athletes, e.g., HC use or access to medical staff, were explored using Chi-square and independent sample *t*-test.

Categorical variables were expressed as frequencies and percentages (%) and continuous variables as means and standard deviations (SD). Support to the athlete, e.g., person to talk to about amenorrhea in an athletic context, was explored with Chi-square tests for differences across athlete level. Differences in aspects of knowledge about female athlete health across athlete level were tested with Chi-square tests, and across age using one-way ANOVA. Effects sizes were calculated using Cohen’s D values for continuous data. The level of statistical significance was set at ≤0.05. All analyses were conducted using the R statistical system version 3.5.2 (R Foundation for Statistical Computing, Vienna, Austria, 2021).

## 3. Results

### 3.1. Support Provided to the Athlete

Of all athletes (*n* = 1086), 58% (*n* = 627) practiced team sports and 42% (*n* = 459) individual sports, 63% (*n* = 679) used hormonal contraceptives and 37% (*n* = 407) were non-users (Table 2). The majority of all athletes (69%, *n* = 747) had a male coach, which was even more common (78%, *n* = 489) among team athletes (Table 2). Most of the athletes had access to medical staff (72%, *n* = 783), with the highest percentage in the top-elite level 90% (*n* = 142). Physiotherapist (47%, *n* = 510) was the most common medical profession to receive support from, and the least common was a gynecologist (3%, *n* = 29). Of the top-elite athletes, 8% (*n* = 13) had access to a gynecologist, compared to 2% (*n* = 8) of the sub-elite athletes (Table 2). For more detailed results on medical support divided by team and individual sports, see Table 2.

**Figure 1:**
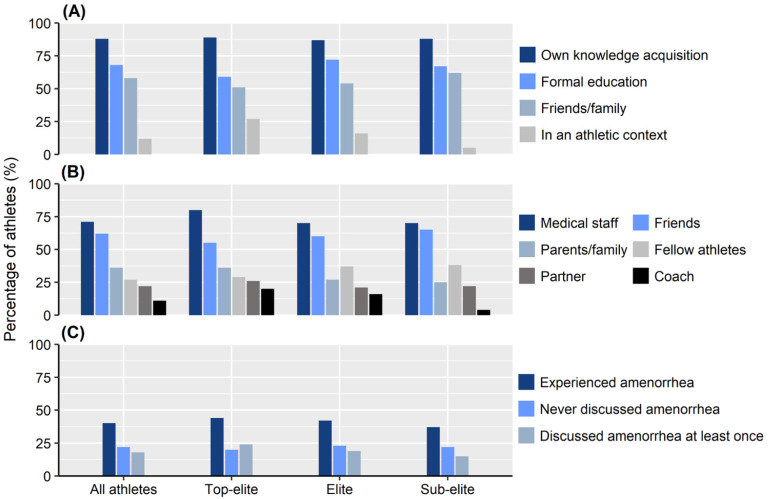
How knowledge about female athlete health was acquired (**A**), person to talk with about female athlete health (**B**) and opportunities to discuss amenorrhea, lasting > 3 months, in the athletic community (**C**).

Sixty percent (*n* = 654) of the athletes had never experienced amenorrhea for more than three months, with a similar distribution across athlete level (*p* = 0.119). About every fifth athlete (22%, *n* = 240) had no one to talk to about amenorrhea in an athletic context (Figure 1). If the athletes had questions about aspects of female athlete health, the majority consulted medical staff (71%, *n* = 774) and/or their friends (62%, *n* = 670), and fewer talked with their coach (11%, *n* = 116), Figure 1. A significantly (*p* = 0.002) higher proportion of athletes with a female coach (55%, *n* = 78) discussed such aspects with their coach, compared to athletes with a male coach (4%, *n* = 28) (Table 3). Top-elite athletes (20%, *n* = 32) consulted their coaches significantly (*p* < 0.001) more frequently about aspects of female athlete health compared to sub-elite athletes (4%, *n* = 23).

### 3.2. Knowledge about Aspects of Female Athlete Health

**Figure 2:**
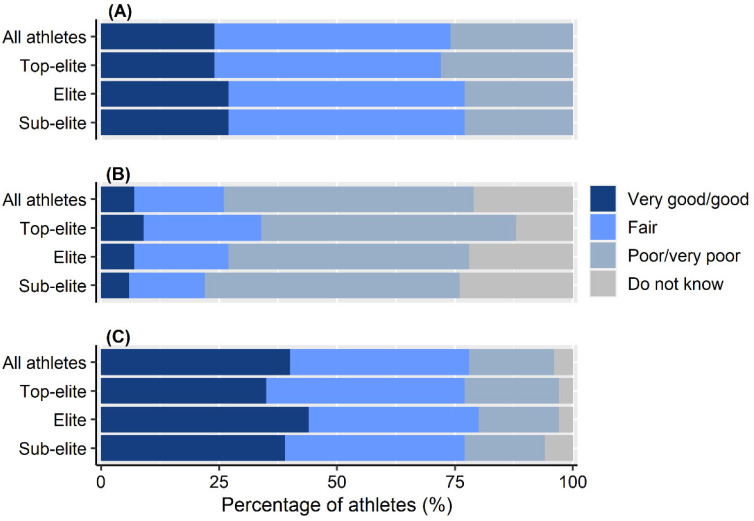
Athletes’ own perceived knowledge about aspects of female athlete health that might influence athletic performance (**A**), their coach’s knowledge in this area (**B**) and whether this subject was taboo in the athletic community (**C**) across athlete level. For “C”, the ordinal scale “*Strongly agree/agree*”, “*Partly agree*”, “*Disagree/strongly disagree*”, “*Do not know*” was used in descending order.

The majority of athletes (53%, *n* = 572) perceived their coach’s knowledge about female athlete health as being poor or very poor, while almost a quarter (24%, *n* = 264) perceived their own knowledge in this area to be good or very good (Figure 2). A significantly (*p* < 0.001) higher proportion of female (30%, *n* = 31) compared to male coaches (5%, *n* = 31) was perceived to have good or very good knowledge (Table 3). Most athletes (88%, *n* = 954) had acquired knowledge about female athlete health on their own, whereas 12% (*n* = 130) had acquired such knowledge in an athletic context, including a significantly (*p* < 0.001) higher proportion of top-elite athletes (27%, *n* = 43) compared to sub-elite athletes (5%, *n* = 26) (Figure 1).

**Figure 3:**
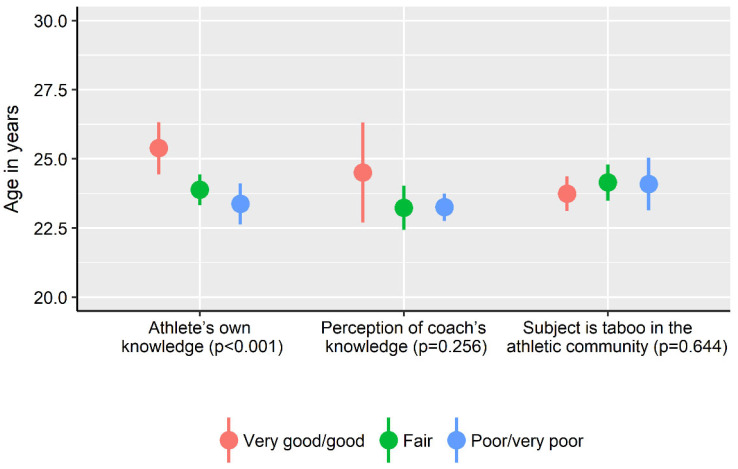
Athletes’ own perceived knowledge about aspects of female athlete health that might influence athletic performance, their coach’s knowledge in this area and whether this subject was taboo in the athletic community across age of the athletes. Confidence intervals (95%) depicted by error bars. *p*-values are based on one-way ANOVA for differences across age for each item. For item “*Subject is taboo in the athletic community*”, the ordinal scale “*Strongly agree/agree*”, “*Partly agree*”, “*Disagree/strongly disagree*” was used in descending order.

Forty percent of the athletes (*n* = 433) strongly agreed or agreed that female athlete health is considered a taboo topic in sports (Figure 2). There were no differences across athlete level of athlete knowledge about female athlete health (*p* = 0.171), athlete perception of their coach’s knowledge (*p* = 0.203) or the proportion of athletes that considered female athlete health to be a taboo topic (*p* = 0.434) (Figure 2). Athletes who considered their own knowledge as very good/good were significantly (*p* < 0.001) older compared to athletes that rated their knowledge as fair or poor (Figure 3), with small effect sizes for age-group differences (Cohen’s d 0.22–0.29). There was no difference (*p* > 0.05) in age between athletes that rated their knowledge as fair compared to poor, the proportion of athletes that perceived female athlete health to be a taboo topic and how the coach’s knowledge was viewed (Cohen’s d 0.01–0.20).

## 4. Discussion

The 1086 athletes who responded to our survey represent the largest sample investigating level of knowledge and communication about the MC, MC-related disturbances, and use of HC in the athletic community. The study included female athletes at top- elite, elite and sub-elite level from 57 different sports divided in team and individual sports. The major finding was that only 24% of the athletes perceived their knowledge about female health to be good or very good. The athletes perceived most of their coaches to have poor/very poor knowledge about female athlete health. Still, the knowledge of female coaches was perceived to be better than that of male coaches. The athletes’ knowledge about female athletes’ health was not acquired in an athletic context, and only 10% discussed such matters with their coach. However, it was significantly more common to discuss female athletes’ health with a female than with a male coach. About three quarters of the athletes partly to strongly agreed that this topic is considered a taboo in the athletic community. The athletes’ performance level was not associated with their own knowledge about female athlete health, their coach’s knowledge in this area or the extent to which they considered this subject to be taboo. Most athletes had access to a medical team, but while the most common profession in the team was a physiotherapist, only three percent had access to a gynecologist.

Only one-fourth of the female athletes in our study felt that they had adequate knowledge concerning the potential effects of the MC and HCs on training and performance, a low proportion that was nonetheless higher than the 8% reported by Solli et al. [2]. Older athletes had more knowledge in this respect, which was most often acquired on their own, from friends and/or through formal education, rather than in connection with sports. Perceived low levels of knowledge have previously been shown to hinder communication between athletes and coaches [2,13].

The unwillingness of the subjects in the present study to discuss the potential effects of the MC and HCs on training and performance with their coaches might therefore be related to their perception that both athletes and coaches had poor knowledge about these matters, which has also been shown in previous studies [2,13]. Another important factor is the high proportion of male coaches in female sport. For example, in the 2020 Olympic Games in Tokyo, 58% of the Swedish participants were women, whereas, according to the Swedish Sports Confederation [21], 74% of the Swedish national team coaches were men. Similarly, in Norway, 86% of the elite-level coaches was reported to be men [22], and 69% of the subjects in the present study had a male coach, which appears to be one reason athletes felt uncomfortable or inexperienced in connection with discussing issues related to female health with their coach [13]. This indicates that educational efforts for athletes and coaches, as well as a greater gender balance among coaches and support teams, are needed to enhance communication.

Previous reports that female athletes do not feel comfortable discussing the MC and HCs and the potential effects on training and performance with their coach [2,5,13] were confirmed in the present study, including 57 different sports. Ten percent of the athletes in the present study discussed such matters with their coach. Additionally, it was considered more comfortable to talk to a female coach, in agreement with the findings by Solli et al. [1]. Of all athletes in the present study, the majority (69%) had a male coach, but the figure was even higher in team sports (78%). Moreover, 80% agreed fully or in part that this subject is a taboo in the athletic community, and almost all had acquired knowledge about menstrual-related symptoms and HCs from friends, relatives, or formal education. Athlete level was not associated with the proportion of athletes experiencing female athlete health being treated as a taboo subject. However, a significantly higher proportion of the top-elite athletes had acquired their knowledge in an athletic context compared to elite and sub-elite athletes. Overall, our observations reinforce, in a large number of respondents, the suggestions by Findlay et al. [2] of support teams to initiate and normalize conversations about the MC, especially in a male-dominated environment.

Instead of turning to the coach for support related to female health issues related to their sporting performance, the female athletes in the present study sought out support from medical staff or friends. Similarly, the respondents in the qualitative study by Findlay et al. [2] reported that they felt more comfortable talking with medical staff about these issues. Most of the subjects (72%) in the present study reported having access to medical staff, especially the group of top-elite athletes. Only 3% had access to a gynecologist and 23% to a physician, whereas access to a physiotherapist was most common (47%). However, access to gynecologist was more common in individual sports (4%) and in the top-elite level (8%), whereas access to physiotherapist was more common in team sports (50) and in the top-elite level (70%). Even if the athlete perceives medical staff to be more comfortable to turn to, limited knowledge about the components and treatment of the female athlete triad has been reported in medical staff [19,20]. This could indicate a lack of knowledge concerning other symptoms related to the MC as well and once again demonstrates the importance of education, also for medical staff.

More than one-fourth of the subjects in the present study reported that they had no one to talk to about amenorrhea in relation to athletic training and performance. This is worrisome, since appropriate treatment can ameliorate both the short- and long-term deleterious consequences of amenorrhea [8]. In this context as well, access to a multi-professional team including, in addition to the coaches, a variety of medical professionals (e.g., a dietician, gynecologist, physician, physiotherapist, and psychologist) is of considerable value [1,13]. The need of support for the athlete was confirmed in our study of more than 1000 athletes, making these results generalizable.

Although surveys such as ours, based on self-assessments, have obvious limitations, we did test our questionnaire in a pilot study and made the modifications required to improve clarity. Since this survey was web-based and distributed via social media and emails, we know neither the total number of receivers nor, consequently, the response rate. Our questionnaire was sent to athletes only, and it would be of considerable interest to compare their responses to those of coaches to the same questions, especially concerning knowledge about the potential influence of the MC and HCs on the training and performance of female athletes. Moreover, it would be of interest to compare the extent of such knowledge displayed by male and female coaches, as well as their willingness to discuss these issues with their athletes.

## 5. Conclusions

In the largest study population investigating knowledge and communication about female athletes’ health to date, most of the examined athletes perceived their own and their coaches’ knowledge concerning the potential influence of the MC and HCs on training and performance as poor or very poor. The athletes perceived female coaches to have more knowledge than men and be easier to talk to about MC and HC issues. However, the majority of the athletes had a male coach. Clearly, the lack of knowledge does not promote discussion about such matters between athletes and coaches. In fact, most athletes perceived MC and HC issues to be taboo, and the support needed is seldom gained through the support systems offered by the sport community. To initiate better communication and optimize the well-being, training and performance of female athletes, there is a need for increased educational efforts in the athletic community. Although most athletes have access to medical support, a multi-professional team including the coach and medical staff (e.g., dietician, gynecologist, physician, physiotherapist, and psychologist) with sufficient knowledge about female health would be highly beneficial for female athletes at all levels.

## Figures and Tables

**Table 1:** The different sports represented, *n* (percentage) of participants in each sport. Athletes from team sports, *n* = 627 (58) and athletes from individual sports, *n* = 459 (42).

**TEAM SPORTS**	**627 (58)**	Roller derby	7 (1)
Soccer	312 (29)	Athletics (discus, javelin)	6 (1)
Handball	243 (22)	Ski jumping	6 (1)
Floorball	42 (4)	Climbing	4 (<1)
Basketball	8 (1)	Rowing	4 (<1)
Ice hockey	8 (1)	Skateboarding	4 (<1)
Volleyball	6 (1)	Weightlifting	4 (<1)
Rugby	4 (<1)	Curling	3 (<1)
Beach volleyball	2 (<1)	Gym training	3 (<1)
American football	1 (<1)	Judo	3 (<1)
Bandy	1 (<1)	Badminton	2 (<1)
_______________________	________	Canoeing (sprint)	2 (<1)
**INDIVIDUAL SPORTS**	**459 (42)**	CrossFit	2 (<1)
Orienteering	94 (9)	Snowboarding	2 (<1)
Cross-country skiing	58 (5)	Ski orienteering	2 (<1)
Swimming	35 (3)	Tennis	2 (<1)
Gymnastics	31 (3)	Equestrian vaulting	2 (<1)
Triathlon	24 (2)	Wrestling	2 (<1)
Powerlifting	19 (2)	Aerobics	1 (<1)
Cycling	17 (2)	Enduro	1 (<1)
Budo	16 (1)	Free skiing	1 (<1)
Alpine skiing	15 (1)	Drill	1 (<1)
Biathlon	15 (1)	Golf	1 (<1)
Figure skating	14 (1)	Kick boxing	1 (<1)
Athletics (sprint/jump)	12 (1)	Rhythmic gymnastics	1 (<1)
Athletics (distance running *)	13 (1)	Shooting	1 (<1)
Canoeing (distance)	9 (1)	Ski cross	1 (<1)
Equestrian	8 (1)	Not indicated	1 (<1)
Archery	7 (1)		

* middle-/long-distance running.

**Table 2:** Background characteristics and support, in terms of sex of main coach and access to medical staff, for the female athletes included in this survey divided into team (*n* = 627) and individual sports (*n* = 459).

	All Athletes (*n* = 1086)			Top-Elite (*n* = 158)			Elite (*n* = 393)			Sub-Elite (*n* = 535)		
	TEAM *n* = 627 (58)	IND *n* = 459 (42)	*p*-Value	TEAM *n* = 30 (19)	IND *n* = 128 (81)	*p*-Value	TEAM *n* = 146 (37)	IND *n* = 246 (63)	*p*-Value	TEAM *n* = 452 (84)	IND *n* = 83 (16)	*p*-Value
Age, mean (SD)	23.6 (6.3)	24.8 (7.4)	0.006	26.2 (0.7)	26.7 (6.4)	0.766	22.6 (5.0)	22.8 (6.2)	0.843	23.7 (6.7)	27.9 (10.0)	<0.001
BMI, mean (SD)	23.6 (2.8)	22.6 (9.0)	<0.001	23.6 (0.5)	22.4 (2.6)	0.013	23.5 (0.6)	22.5 (2.6)	<0.001	23.5 (8.7)	23.6 (3.6)	0.805
Sex of coach, *n* (%)												
Male	489 (78)	258 (56)	<0.001	25 (86)	86 (67)	0.082	113 (77)	140 (57)	<0.001	337 (75)	50 (60)	0.007
Female	78 (12)	65 (14)	0.407	3 (10)	12 (9)	0.916	21 (15)	40 (16)	0.767	54 (12)	14 (17)	0.216
Male and female	45 (7)	44 (9)	0.191	1 (3)	12 (9)	0.278	9 (6)	22 (9)	0.330	35 (8)	10 (12)	0.194
No main coach	15 (2)	93 (20)	<0.001	0	19 (15)	n.a.	2 (1)	44 (18)	<0.001	20 (4)	9 (11)	0.021
Access to medical staff *, *n* (%)												
Medical staff, overall	453 (72)	331 (72)	0.961	29 (97)	112 (88)	0.145	133 (91)	181 (74)	<0.001	292 (65)	40 (48)	0.005
Dietician	61 (10)	110 (24)	<0.001	17 (57)	62 (48)	0.417	21 (14)	46 (18)	0.280	24 (5)	4 (5)	0.854
Gynecologist	9 (1)	20 (4)	0.003	0	13 (10)	n.a.	3 (2)	7 (3)	0.636	8 (2)	1 (1)	0.713
Physician	109 (17)	142 (31)	<0.001	25 (83)	72 (56)	0.006	40 (27)	63 (26)	0.680	44 (10)	8 (10)	0.978
Physiotherapist	315 (50)	195 (42)	0.011	30 (100)	81 (63)	<0.001	101 (69)	101 (41)	<0.001	183 (40)	16 (19)	<0.001
Use of HC	437 (70)	242 (53)	<0.001	15 (50)	57 (45)	0.588	107 (73)	128 (52)	<0.001	316 (70)	41 (49)	<0.001

BMI = body mass index, TEAM = team player athlete, IND = individual competitive athlete, HC = hormonal contraceptives. * Medical staff includes chiropractor, dietician, gynecologist, mental coach, masseur, naprapathy, physiotherapist, physician, and physical trainer. n.a. = not applicable.

**Table 3:** Opportunities to talk about amenorrhea (lasting > 3 months), perception of coach’s knowledge about aspects of female athlete health and whether this subject is considered to be taboo in the athletic community, by sex of coach. *p*-values are based on Chi-square tests.

	Male Coach (*n* = 747)	Female Coach (*n* = 143)	*p*-Value
Do talk about female athlete health with their coach, *n* (%)	28 (4)	78 (55)	0.002
Opportunity to discuss amenorrhea, *n* (%)			
Never experienced amenorrhea	155 (21)	30 (21)	0.559
Discussed amenorrhea at least once	143 (19)	22 (15)	
Experienced amenorrhea	298 (40)	52 (36)	
Perception of coach’s knowledge, *n* (%)			
Very good/good	31 (5)	31 (30)	<0.001
Fair	123 (19)	48 (46)	
Very poor/poor	484 (76)	25 (24)	
Female athlete health is taboo in the athletic community, *n* (%)			
Strongly agree/agree	293 (41)	58 (42)	0.948
Partly agree	286 (40)	53 (39)	
Disagree/strongly disagree	138 (19)	26 (19)	

## Data Availability

The data presented in this study are available on request from the corresponding author.
